# MRI contributes to accurate and early diagnosis of non-radiographic HLA-B27 negative axial spondyloarthritis

**DOI:** 10.1186/s12967-021-02959-3

**Published:** 2021-07-09

**Authors:** Chun-Chi Lu, Guo-Shu Huang, Tony Szu-Hsien Lee, En Chao, Hsiang-Cheng Chen, Yong-Si Guo, Shi-Jye Chu, Feng-Cheng Liu, San-Yuan Kao, Tsung-Yun Hou, Chen-Hung Chen, Deh-Ming Chang, Sin-Yi Lyu

**Affiliations:** 1 Division of Rheumatology/Immunology/Allergy, Department of Internal Medicine, Tri-Service General Hospital, National Defense Medical Center, Taipei, Taiwan; 2 Department of Pathology, University of Washington, Seattle, USA; 3 Department of Radiology, Tri-Service General Hospital, National Defense Medical Center, National Defense Medical Center, National Defense Medical Center, No. 100, Zhengrong St., Zhongzheng Dist, Keelung City, 202 Taiwan; 4 Department of Health Promotion and Health Education, National Taiwan Normal University, Taipei, Taiwan; 5 Tri-Service General Hospital Songshan Branch, Taipei, Taiwan; 6 Division of Rheumatology, Department of Internal Medicine, Taipei Tzu Chi Hospital, Taipei, Taiwan; 7 Division of Allergy, Immunology, Rheumatology, Department of Internal Medicine, Taipei Veteran General Hospital, Taipei, Taiwan; 8 Division of Radiology, Tri-Service General Hospital, National Defense Medical Center, Keelung branch, Taipei, Taiwan

**Keywords:** Magnetic resonance imaging, Spondyloarthritis, Joint space widening, Bone marrow edema

## Abstract

**Background:**

Nonradiographic axial spondyloarthropathies (nr-axSpA) are diagnosed by the absence of radiographic sacroiliitis and the presence of bone marrow edema (BME) on magnetic resonance imaging (MRI). According to the classification criteria of the international Assessment of Spondyloarthritis Society (ASAS), structural changes to sacroiliac joints (SIJs) on MRI cannot be used as criteria in the absence of BME. However, less than half the Asian patients with clinically active axSpA show BME. The incidence of human leukocyte antigen (HLA)-B27 is low in Asian populations, which makes it more difficult to identify nr-axSpA. We used MRI to evaluate the structural damage to SIJs in patients with nr-axSpA with and without BME with the aim of identifying the best methodology for accurate diagnosis, especially in populations with less common BME and HLA-B27.

**Methods:**

One hundred three patients with inflammatory back pain were included in this prospective study. No patient’s radiograph met the definition of positive modified New York criteria. BME and structural damage to SIJ including sclerosis and erosion were assessed independently on coronal and axial short-tau inversion recovery and T1-weighted spin echo MRI scans by two well-trained musculoskeletal radiologists using the Spondyloarthritis Research Consortium of Canada (SPARCC) score. Demographics of patients were collected. Disease characteristics and structural damage were analyzed in patients with and without BME on SIJ MRI. Receiver operating characteristic (ROC) curve analysis was used to assess the diagnostic performance of structural damage.

**Results:**

All individuals in the cohort had at least one abnormal finding on SIJ MRI, including BME or structural damage; 36 of 103 patients had BME. We identified a significant positive correlation between SPARCC scores and severe erosion assessed by focal joint space widening (fJSW) (*p* = 0.001) in these 36 patients. Fifty-eight of the 103 enrolled patients fulfilled the ASAS criteria for nr-axSpA in the either absence or presence of BME. Of these 58 patients, 57 and 19 had erosions or fJSW, respectively, and the presence of BME was significantly correlated with fJSW (phi score of 0.319 and *p* = 0.015). We demonstrated a significant positive correlation between fJSW and either the presence or the severity of BME in patients with nr-axSpA who met the ASAS definition. There was a positive correlation between BME and fJSW across the whole study cohort (phi score of 0.389; *p* < 0.001). The area under the ROC curve (AUC) for fJSW on SIJ MRI was 0.736, *p* < 0.001. In both HLA-B27-positive and -negative groups, BME was more common in the presence of fJSW (phi scores of 0.370 and 0.377, *p* = 0.018 and 0.003, respectively) and SPARCC scores were higher in patients with fJSW (*p* < 0.001 and *p* = 0.005). We also identified a positive correlation between fJSW and BME in patients with nr-axSpA and normal serum levels of C-reactive protein (phi score of 0.362 and *p* = 0.001).

**Conclusion:**

Structural damage detected on SIJ MRI, sclerosis, erosions and fJSW may be present in patients without detectable inflammation on SIJ MRI. However, fJSW is significantly correlated with the severity of inflammation seen on SIJ MRI, which contributes to the accurate diagnosis of nr-axSpA, and it could be used as an alternative diagnostic test for nr-axSpA in the general population, especially for those who do not carry the HLA-B27 gene, Asian patients without BME, or patients with normal serum inflammatory biomarkers.

## Background

Spondyloarthropathies (SpA) are a group of chronic inflammatory rheumatic diseases that share overlapping features such as sacroiliitis, enthesitis, extra-articular manifestations including acute anterior uveitis, psoriasis, and inflammatory bowel disease (IBD), HLA-B27 positivity and familial clustering [1–3]. Axial SpA (axSpA), which comprises a spectrum of symptoms of chronic inflammatory back pain (IBP), can be diagnosed as either radiographic axSpA, e.g., ankylosing spondylitis (AS), or nonradiographic axSpA (nr-axSpA), depending on the presence or absence of radiographic sacroiliitis, respectively [4, 5]. AxSpAs include inflammation of the sacroiliac joints (SIJs), facet joints, and spinal entheses, which result in IBP, fatigue, stiffness, and ankyloses [6]. The classification criteria of the ASAS have been widely used for diagnosis of axSpA [7, 8].

Magnetic resonance imaging (MRI) is able to detect both inflammatory lesions, such as bone marrow edema (BME), and structural damage, such as sclerosis, erosions, ankylosis, fat metaplasia and backfill in the SIJs and spine, in patients with radiographic axSpA. Furthermore, MRI is capable of detecting inflammatory lesions before the development of structural changes in SIJs that are detectable on radiography or computed tomography (CT) [7–12]. The benefits of SIJ MRI are that the high soft tissue and bone marrow contrast facilitates detection of BME, which helps to confirm the diagnosis of nr-axSpA. SIJ MRI has been included in the imaging criteria in the ASAS classification criteria for axSpA [13, 14]. However, the limitations of SIJ MRI include the unpredictable interval between the development of structural damage visible on radiographs and the clinical diagnosis [15–19].

Experts generated the ASAS definition of axSpA based only on the detection of BME on SIJ MRI, while structural damage to SIJs has not yet been included [7, 8]. However, BME can be found in healthy people, running enthusiasts, and women postpartum [20–23]. Remission of bone marrow inflammation induces structural damage including fat metaplasia [10, 11]. Although bone erosions can be repaired by backfilling [24], there is no direct evidence indicating that BME induces bone erosion. Whether and how BME detected on SIJ MRI is relevant to bone erosion is unknown. Thus, the question of whether structural damage should be added to the ASAS definition remains controversial [12, 24]. The literature indicates that SIJ MRI can detect bone erosion early in the disease course [25]. Previous data indicated that fewer than half of axSpA patients were positive for active sacroiliitis on SIJ MRI [9, 26–28], which is consistent with our real-world experience. In addition, in patients with early-phase axSpA, HLA-B27 is associated with more prominent inflammation on SIJ MRI, whereas common serum inflammatory biomarkers, such as C-reactive protein (CRP) and erythrocyte sedimentation rate (ESR), are incapable of predicting early sacroiliitis. Whether HLA-B27 is associated with structural damage seen on SIJ MRI remains unknown. All of these factors contribute to the difficulty in defining nr-axSpA in patients of ethnicities that have a lower prevalence of BME on SIJ MRI and of HLA-B27. In the clinic, we noticed that both HLA-B27-positive and -negative patients with chronic IBP often did not have abnormal serum levels of CRP or ESR, even those who demonstrated multiple clinical characteristics of axSpA; this led to underestimation of the prevalence and misdiagnosis of nr-axSpA.

The goals of this study were first, to demonstrate the incidence of BME and erosions detected on SIJ MRI in the local population with clinical IBP and nr-axSpA. Second, we aimed to identify whether erosion detected on SIJ MRI contributes to an accurate diagnosis of nr-axSpA either in the presence or absence of HLA-B27; we hypothesized that erosion would be associated with inflammation seen on SIJ MRI and should be capable of being applied as a diagnostic tool for axSpA. Third, we aimed to determine the best imaging modality for an accurate diagnosis of nr-axSpA in the absence of HLA-B27. Therefore, the aim of this study was to investigate the diagnostic utility of erosion and BME on SIJ MRI, which could be useful in patients of certain ethnicities for accurate and early diagnosis of nr-axSpA.

## Methods

### Patients

**Fig. 1:**
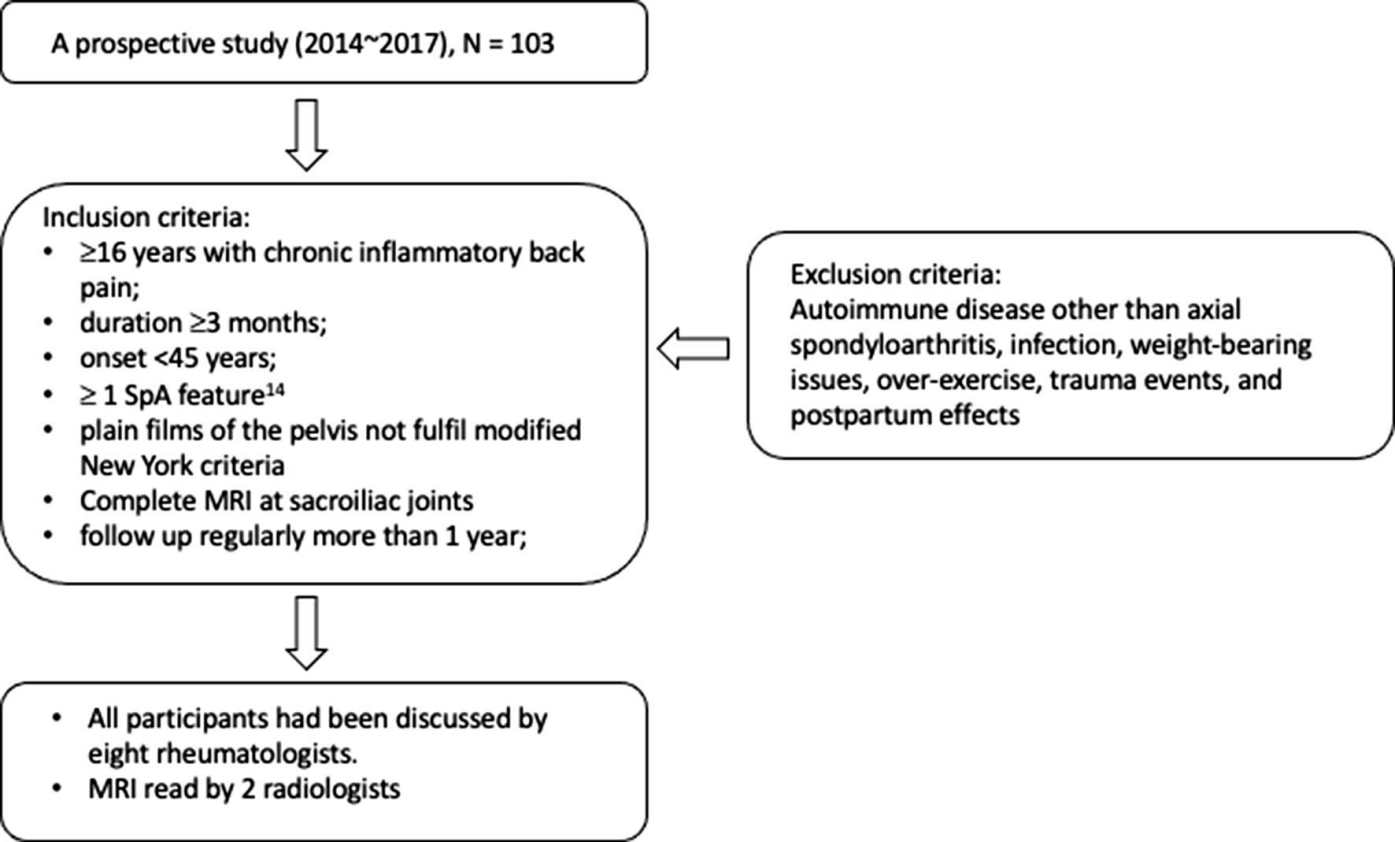
Flow chart of enrolled participants

This prospective cohort study was conducted from 2014 to 2017 and all patients were Taiwanese. Patients  ≥ 16 years with chronic IBP (duration ≥ 3 months, onset  < 45 years, origins unknown) and morning stiffness were recruited into the cohort in the clinics of the division of rheumatology at a university medical center (Fig. 1). This study was approved by the Institutional Review Board of Tri-Service General Hospital (No. 2–106-05–106). Autoimmune diseases other than axSpA, infection, weight-bearing issues, overexercise, traumatic events, and postpartum effects were excluded. Patients were examined by rheumatologists for the presence of features of AS according to the ASAS criteria [29]. The imaging arm of the AS criteria includes radiographic sacroiliitis [modified New York criteria (mNY)] [30], or sacroiliitis on SIJ MRI [14, 31]. Clinical evaluation of disease activity included the Bath Ankylosing Spondylitis Disease Activity Index (BASDAI) and the Ankylosing Spondylitis Disease Activity Scores (ASDAS) [32, 33]. All participants were presented and discussed on the basis of these examinations by two experienced radiologists and eight rheumatologists with an interest and experience in axSpA, conventional radiographs and SIJ MRI. Medical records of all patients were collected from the hospital information system and reviewed. Inclusion and exclusion criteria are illustrated in Fig. 1.

### MRI image analysis

**Fig. 2:**
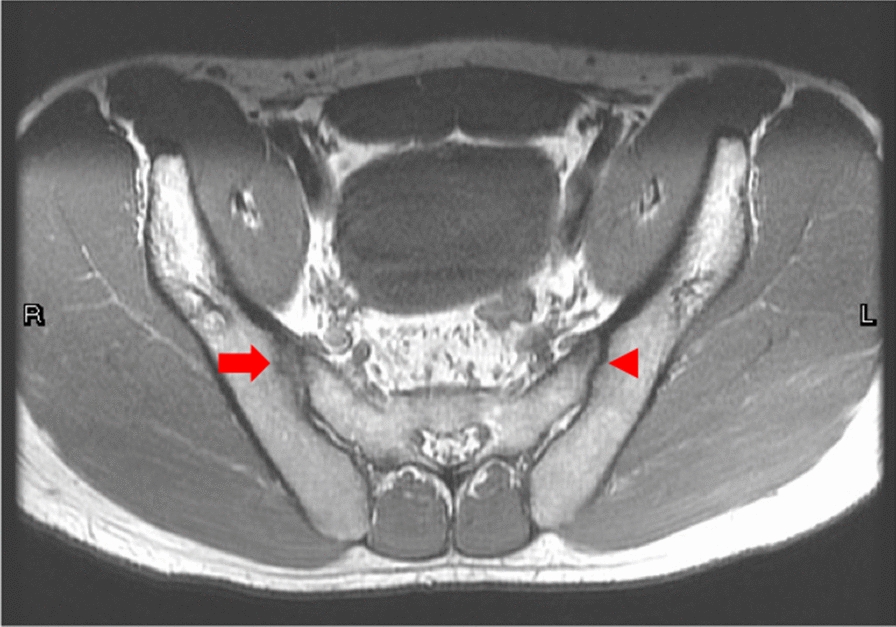
Severe bone erosions at the right sacroiliac joint led to focal joint space widening (red arrow), while the left sacroiliac joint did not reveal significant joint erosions (red arrowhead)

Plain films of the pelvis of all enrolled patients were performed in anteroposterior view. Patients in this study underwent SIJ MRI performed using a 1.5 T scanner, with the patient in the supine position using a high-resolution body phased-array coil. Multiple sequences (coronal and axial T1-weighted spin echo (T1WSE), and coronal and axial short-tau inversion recovery (STIR)) were performed with a slice thickness of 4 mm. The SIJ MRIs were acquired in an oblique coronal plane. Coronal imaging was performed in the oblique coronal plane oriented along the long axis of the sacrum, and axial imaging was performed in the oblique axial plane perpendicular to the oblique coronal plane [34, 35]. BME was defined as detection of a hyperintense signal on coronal STIR imaging, which must be homogeneous and extend more than 1 cm in depth from the joint surface, when at least one lesion was present in at least two consecutive slices or at least two lesions were found in one slice [7, 14, 31]. The Spondyloarthritis Research Consortium of Canada (SPARCC) scoring system was adopted to assess the severity of BME seen on SIJ MRI [25]. Structural damage visible on SIJ MRI was evaluated based on ASAS and Outcome Measures in Osteoarthritis definitions and their updates [24, 31, 36–38]. Erosion was defined as a break in the cortical bone of the iliac or sacral bones, which appeared dark on both T1WSE and STIR sequences [39]. We noticed that some patients in the cohort showed severe bone erosion at the SIJ, which lead to focal joint space widening (fJSW) (Fig. 2), which was not noted or confirmed on conventional radiographs. Two well-trained musculoskeletal radiologists (Drs. GS Huang and SY Lyu) independently scored all MRIs with both sequences viewed simultaneously. The reading process was completely blinded: readers had no knowledge of patient characteristics or other clinical and imaging data. Interreader agreement was defined as agreement between the two readers for the same image. For intrareader reliability, the MRI interpretation was assessed using Fleiss kappa coefficients [40–42]. Kappa values of both SIJs were compared to determine the concordance of positivity on MRI and were higher than 0.6.

### Statistical analyses

Patient demographic information and characteristics are summarized as frequencies and percentages for categorical values, and are presented as the mean and standard deviation, as appropriate, for continuous variables. An association between the severity of BME and structural damage on SIJ MRI was calculated using Spearman’s rho scores for small populations. Receiver operating characteristic (ROC) curve analysis was used to assess the diagnostic performance of structural damage detected on SIJ MRI. A discriminatory power of 0.7–0.79 is considered fair performance [43]. Differences between categories of structural damage and the effects of the presence or absence of HLA-B27 on grades of BME on SIJ MRI (SPARCC scores) and ASDAS-CRP scores were calculated using a Mann–Whitney *U* test. The strength of associations between categories of structural damage and the presence or absence of HLA-B27 were calculated using Pearson chi-square and phi coefficient scores. Analyses were performed using SPSS for Windows, version 15.0.1.1 (SPSS Inc., Chicago, IL).

## Results

The demographics and disease activity scores of patients are listed in Table 1. All 103 participants were clinically suspected of having axSpA and none of them met the mNY criteria on conventional radiographs [5, 14, 30]. Thirty-six patients (group 1) had “positive SIJ MRIs” [MRI( +)/mNY(–)], ≥ 1 AS features and fulfilled the ASAS criteria for axSpA [31]. Among the 67 MRI(–)/mNY(–) participants, there was a subgroup of 22 HLA-B27-positive patients with ≥ 2 additional clinical AS features (group 2) [MRI(–)/mNY(–)/HLA-B27( +)], who met the ASAS criteria for axSpA. Forty-five patients were classified as a triple-negative subgroup [MRI(–)/mNY(–)/HLA-B27(–)] (group 3). Participants in group 3 had a high possibility of nr-axSpA because of the presence of both IBP and a good response to NSAIDs, plus at least one clinical feature of AS (psoriasis, dactylitis, uveitis, heel enthesitis, peripheral arthritis, or positive family history of AS). A complete and detailed description of the method for participant classification is presented in Table 1.

**Table 1:** Demographic of 103 nr-axSpA subjects with chronic inflammatory back pain and morning stiffness

	Age (yr)	Gender male (%)	BASDAI	ASDAS-CRP	ASDAS-ESR	Abnormal serum CRP (%)	Abnormal serum ESR (%)	SPARCC scores	Structural damage of MRI-SIJs				
									Sclerosis	Erosion	fJSW	DP^a^	TP^b^
Group 1 (n = 36)	31.3 ± 11.99	75%	2.47 ± 1.50	2.19 ± 0.66	1.90 ± 0.81	30.6%	33.3%	6.73 ± 7.73	77.8%	97.2%	44.4%	33.3%	30.6%
Group 2 (n = 22)	31.59 ± 12.51	86.4%	1.98 ± 0.82	1.86 ± 0.85	1.60 ± 0.60	22.7%	18.2%	NA	40.9%	100%	13.6%	27.3%	13.6%
Group 3 (n = 45)	30.09 ± 13.78	66.7%	2.36 ± 0.89	2.03 ± 0.59	1.73 ± 0.59	20%	24.4%	NA	51.1%	93.3%	8.9%	37.8%	8.9%
Group 1 + 2^c^ (n = 58)	31.41 ± 12.08	79.3%	2.28 ± 1.27	2.06 ± 0.75	1.79 ± 0.75	27.6%	27.6%	4.83 ± 6.85	63.8%	98.3%	32.8%	32.8%	31.0%
Group 1 + 2 + 3 (n = 103)	32.15 ± 12.81	73.8%	2.32 ± 1.12	2.05 ± 0.68	1.76 ± 0.68	24.3%	26.2%	2.49 ± 5.46	58.3%	96.1%	22.3%	35.0%	21.4%

*BASDAI* Bath Ankylosing Spondylitis Disease Activity Index, *ASDAS* Ankylosing Spondylitis Disease Activity Scores, *CRP* C-reactive protein, *ESR* erythrocyte sedimentation rate, *BME* bone marrow edema, *MRI* magnetic resonance imaging, *SIJ* sacroiliac joint, *SPARCC* Spondyloarthritis Research Consortium of Canada, *fJSW* focal joint space widening, *ASAS* Assessment of Spondyloarthritis international Society, *nr-axSpA* nonradiographic spondyloarthritis; Group 1, (MRI( +)/ mNY(−)); Group 2, [MRI(−)/ mNY(−)/ HLA-B27( +)]; Group 3, [MRI(−)/ mNY(-)/ HLA-B27(−)];* NA* not applicable; ^a^DP, double positive, dual presence of sclerosis and erosion on SIJ MRI; ^b^TP, triple positive, simultaneous presence of sclerosis, erosion, and fJSW on MRI-SIJs; ^c^group 1 + 2, ASAS defined nr-axSpA

### Imaging of MRI SIJs in subgroups of nr-axSpA: correlation between inflammation and structural damage

None of the participants’ radiographs met the mNY criteria for AS. The 36 patients in group 1 had positive SIJ MRIs [MRI( +)/mNY(–)]. Their average BME SPARCC scores were 6.73 ± 7.725 (Table 1). In this group, 97.2% and 44.4% of patients had erosions or fJSW, respectively. There were significant positive correlations between SPARCC scores and sclerosis, fJSW, or the triple-positive of sclerosis, erosion, and fJSW (*p* = 0.001, 0.004, and 0.001, respectively, Table 2). HLA-B27-positive subjects had higher mean SPARCC scores than those without HLA-B27 (*p* = 0.015).

**Table 2:** Difference between structural damage and the presence/absence of HLA-B27 on the SPARCC scores in nr-axSpA subjects (Mann–Whitney U test)

	SPARCC scores nr-axSpA (n = 103)	SPARCC scores HLA-B27( +) (n = 41)	SPARCC scores HLA-B27(–) (n = 62)	SPARCC scores BME( +)/mNY(–) (group 1: n = 36)
Sclerosis ( +) vs. (–) Numbers (%) *p* value	3.98 vs. 0.40 60 (58.3%) vs. 43 (41.7%) < 0.001***	5.77 vs. 0.67 26 (63.4%) vs. 15 (36.6%) 0.002**	2.62 vs. 0.25 34 (54.8%) vs. 28 (45.2%) 0.161	8.54 vs. 2.13 28 (77.8%) vs. 8 (22.2%) 0.001**
Erosion ( +) vs. (–) Numbers (%) *p* value	2.57 vs. 0.50 99 (96.41%) vs. 4 (4.6%) 0.663	3.90 [41] vs. 0.00 41 (100%) vs. 0 NA	1.62 vs. 0.50 58 (93.5%) vs. 4 (6.5%) 1.000	7.26 vs. 2.00 35 (97.2%) vs. 1 (2.8%) 0.500
fJSW ( +) vs. (–) Numbers (%) *p* value	7.52 vs. 1.04 23 (22.3%) vs. 80 (77.7%) < 0.001***	8.58 vs. 1.97 12 (29.3%) vs. 29 (70.7%) < 0.001***	6.36 vs. 0.51 11 (17.7%) vs. 51 (82.3%) 0.005**	10.81 vs. 4.15 16 (44.4%) vs. 20 (55.6%) 0.004**
DP^a^ (sclerosis/erosion) vs. (–) Numbers (%) *p* value	2.82 vs. 1.86 67 (60%) vs. 36 (40%) 0.942	4.19 vs. 3.36 27 (65.9%) vs. 14 (34.1%) 0.734	1.91 vs. 0.91 40 (64.5%) vs. 22 (35.5%) 0.422	8.22 vs. 5.15 23 (63.9%) vs. 13 (36.1%) 0.474
TP^b^ (sclerosis/erosion/fJSW) vs. (–) Numbers (%) *p* value	7.82 vs. 1.04 22 (21.4%) vs. 81 (78.6%) < 0.001***	8.58 vs. 1.97 12 (29.3%) vs. 29 (70.7%) 0.001**	6.90 vs. 0.52 10 (16.4%) vs. 52 (83.6%) 0.005**	11.47 vs. 4.00 15 (41.7%) vs. 21 (58.3%) 0.001**

Scores were evaluated by two experienced radiologists by consensus. For intrareader reliability, the MRI interpretation was measured with Fleiss kappa coefficients. *SPARCC* Spondyloarthritis Research Consortium of Canada,* fJSW* focal joint space widening, *NA* not applicable

^a^DP, double positive, dual presence of sclerosis and erosion on SIJ MRI

^b^TP, triple positive, simultaneous presence of sclerosis, erosion, and fJSW on MRI-SIJs

**p value < 0.01

***p value < 0.001

In group 2 [MRI(–)/mNY(–)/HLA-B27( +)], we identified 100% and 13.6% of patients with erosions, and fJSW, respectively (Table 1). In the whole cohort of this study, 58 patients (from groups 1 and 2) fulfilled the ASAS classification criteria; 98.3%, and 32.8% of these patients had erosions or fJSW, respectively (Table 1). With respect to the inflammation and structural damage in these 58 ASAS-defined nr-axSpA subjects, BME on SIJ MRI was moderately correlated with sclerosis and fJSW (phi scores of 0.372 and 0.319, *p* = 0.005 and 0.015, respectively). BME was positively correlated with triple-positive signs of structural damage including sclerosis, erosions, and fJSW (TP sign) on SIJ MRI (phi score of 0.294, *p* = 0.025). Among 45 patients in group 3; 93.3% and 8.9% had erosions and fJSW, respectively (Table 1). Thus, we demonstrated a significant positive correlation between either the presence or the severity of BME and fJSW in patients with nr-axSpA that met the ASAS definition. Moreover, a similar positive correlation was found when patients were characterized by simultaneous triple-positivity for sclerosis, erosions, and fJSW.

**Fig. 3:**
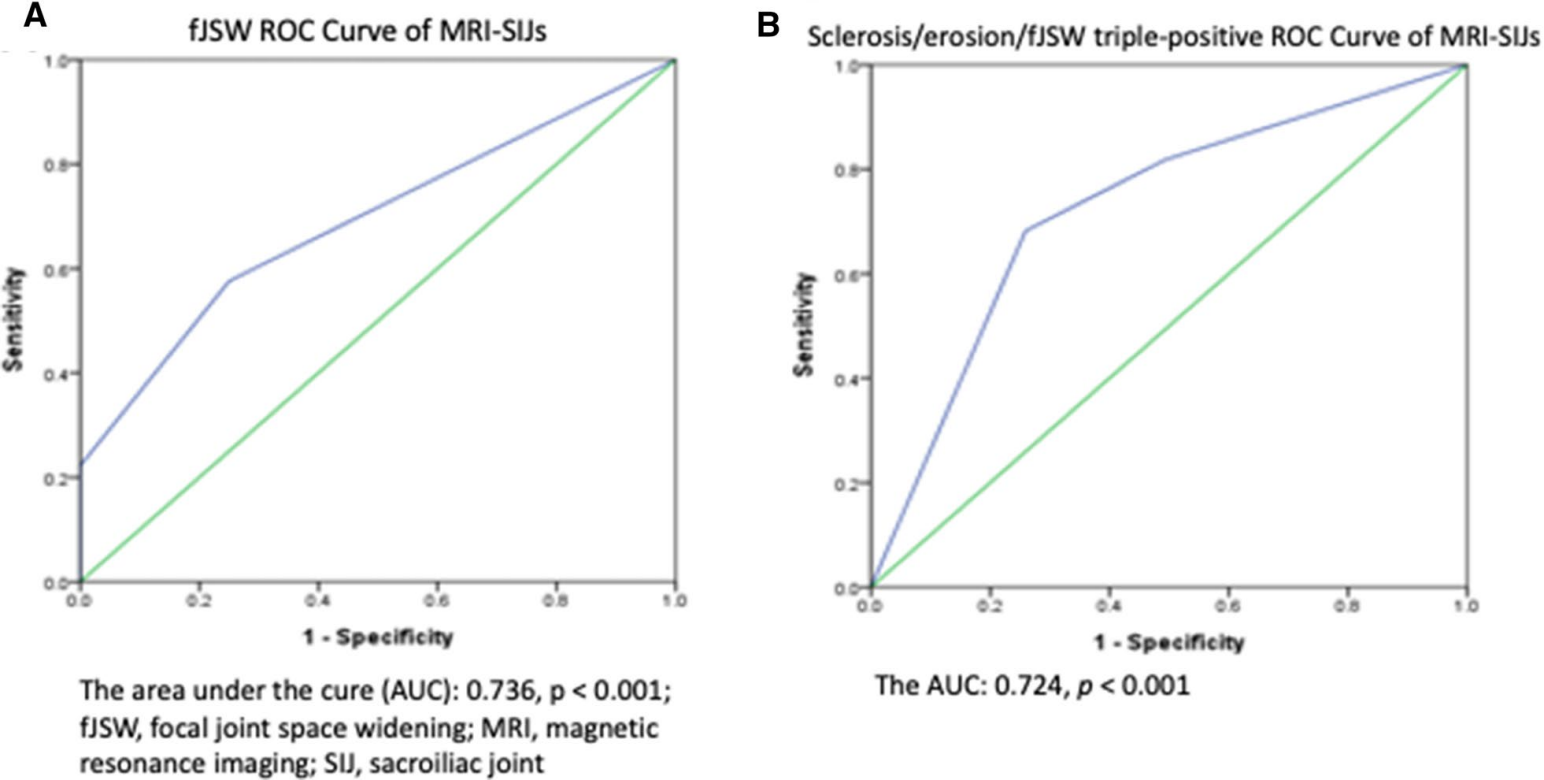
The area under the curve (AUC) for fJSW was 0.736, *p* < 0.001 (**A**); that for triple-positivity for sclerosis, erosion, and fJSW was 0.724, *p* < 0.001 (**B**).* fJSW* focal joint space widening, * MRI* magnetic resonance imaging,* SIJ* sacroiliac joint

All patients in the study cohort had clinical IBP and at least one abnormal finding on SIJ MRI, including BME or structural damage. Sixteen of 23 patients with fJSW had BME, while 20 of 80 patients without fJSW had BME (*p* < 0.001). Twenty-two patients had triple-positivity for presence of sclerosis, erosions, and fJSW, and 15 had BME on SIJ MRI. Of the other 81 patients, 22 had BME (*p* < 0.001). There was a positive correlation between BME and sclerosis or fJSW (phi scores of 0.290 and 0.389; *p* = 0.003 and < 0.001, respectively). There was also a positive correlation between BME and the triple-positive for presence of sclerosis, erosions, and fJSW (phi score of 0.363; *p* < 0.001; Table 3). The areas under the ROC curve (AUC) for fJSW and triple-positivity for presence of sclerosis, erosions, and fJSW were 0.736 and 0.724, both *p* < 0.001; Fig. 3.

**Table 3:** Associations and strengths between the presence of BME and structural damage of MRI-SIJs in 103 nr-axSpA subjects (Subject numbers, Pearson Chi-Square & phi scores)

	Sclerosis		Erosion		fJSW		DP		TP	
	( +)	(–)	( +)	(–)	( +)	(–)	( +)	(–)	( +)	(–)
BME ( +) (%)	28/60 (47.7%)	8/43 (18.6%)	35/99 (45.4%)	1/4 (25%)	16/23 (69.6%)	20/80 (25%)	13/36 (36.1%)	23/67 (34.3%)	15/22 (68.2%)	21/81 (25.9%)
*p* value	0.004**		0.670		< 0.001***		0.856		< 0.001***	
phi scores	0.290		0.670		0.389		0.018		0.363	

*BME* bone marrow edema,* MRI* magnetic resonance imaging, *SIJ* sacroiliac joint, *nr-axSpA* nonradiographic spondyloarthritis, *JSW* joint space widening, *DP* double positive, dual presence of sclerosis and erosion on SIJ MRI, *TP* triple positive, simultaneous presence of sclerosis, erosion, and fJSW on MRI-SIJs

**p value < 0.01

***p value < 0.001; phi scores were used for association measure between binary variables

In addition, there was a positive correlation between SPARCC scores and sclerosis (0.4 in sclerosis-negative subjects vs. 3.98 in sclerosis-positive subjects, *p* < 0.001) for the entire cohort. We also identified a positive correlation between SPARCC scores and fJSW (1.04 in fJSW-negative subjects vs. 7.52 in fJSW-positive subjects, *p* < 0.001). There was a positive correlation between SPARCC scores and the triple-positive for presence of sclerosis, erosions, and fJSW (*p* < 0.001) (Table 4). The data indicate that not only is BME correlated with fJSW, but also that the severity of BME is positively correlated with fJSW. All patients in the cohort with fJSW had higher ASDAS-CRP scores (*p* = 0.01, respectively) than those without fJSW. Subjects with both sclerosis and erosions had higher ASDAS-CRP scores (*p* = 0.04). Subjects who were triple-positive for sclerosis, erosions, and fJSW had significantly higher ASDAS-CRP scores (*p* = 0.02, respectively) (Table 4).

**Table 4:** Difference between structural damage and the presence/absence of HLA-B27 on the SPARCC scores or ASDAS-CRP level in nr-axSpA subjects (Mann–Whitney U test)

Presence ( +) vs. absence (–) of structural damage	HLA-B27( +) n = 41	HLA-B27(–) n = 62	nr-axSpA n = 103
SPARCC scores			
Sclerosis ( +) vs. (–) *p* value	5.77 vs. 0.67 0.002**	2.62 vs. 0.25 0.161	3.98 vs. 0.40 < 0.001***
Erosion ( +) vs. (–) *p* value	3.90 vs. 0.00 NA^c^	1.62 vs. 0.50 1	2.57 vs. 0.50 0.663
JSW ( +) vs. (–) *p* value	8.58 vs. 1.97 < 0.001***	6.36 vs. 0.51 0.005**	7.52 vs. 1.04 < 0.001***
DP^a^ ( +) vs. (–) *p* value	4.19 vs. 3.36 0.734	1.90 vs. 0.91 0.422	2.82 vs. 1.86 0.942
TP^b^ ( +) vs. (–) *p* value	8.58 vs. 1.97 0.001**	6.90 vs. 0.52 0.005**	7.82 vs. 1.04 < 0.001***
ASDAS-CRP scores			
Sclerosis ( +) vs. (–) *p* value	2.04 vs. 2.02 0.678	2.09 vs. 2.03 1	2.04 vs. 2.06 0.848
Erosion ( +) vs. (–) *p* value	2.03 vs. 0.00 NA	2.23 vs. 2.04 0.308	2.23 vs. 2.04 0.270
fJSW ( +) vs. (–) *p* value	2.38 vs. 1.89 0.034*	2.31 vs. 2.00 0.074	2.34 vs. 1.96 0.010*
DP^a^ ( +) vs. (–) *p* value	2.18 vs. 1.76 0.108	2.14 vs. 1.91 0.199	2.15 vs. 1.85 0.050
TP^b^ ( +) vs. (–) *p* value	2.38 vs. 1.89 0.034*	2.27 vs. 2.02 0.173	2.33 vs. 1.97 0.020*

*BME* bone marrow edema, *SPARCC* Spondyloarthritis Research Consortium of Canada, *ASDAS* Ankylosing Spondylitis Disease Activity Scores, *CRP* C-reactive protein,* fJSW* focal joint space widening, *nr-axSpA* nonradiographic spondyloarthritis, *NA* not applicable

^a^DP, double positive, dual presence of sclerosis and erosion on MRI-SIJs

^b^TP, triple positive, simultaneous presence of sclerosis, erosion, and fJSW on MRI-SIJs

^c^All HLA-B27 ( +) patients have erosions on SIJ MRI

*p value < 0.05

**p value < 0.01

***p value < 0.001

### Correlations between disease activity, inflammatory biomarkers, and SIJ MRI

Our data showed that 30.6%, 22.7%, and 20% of subjects in groups 1, 2, and 3, respectively, had increased serum CRP levels (Table 1), and that 25 of 36 patients in group 1 had BME and normal serum CRP levels. This indicated that symptomatic nr-axSpA subjects with acute bone marrow inflammation could still have normal serum CRP levels, and mirrored the other challenge of accurately diagnosing nr-axSpA in patients with IBP but normal CRP. We also demonstrated the same phenotype in this cohort using serum ESR levels (Table 1). In our clinical practice we have noticed that some AS patients who meet the mNY criteria have normal serum CRP and ESR, as do some patients with nr-axSpA who meet the ASAS criteria. According to the policy of the health insurance system in some countries, these radiographic and nr-axSpA patients cannot be prescribed biological agents that might predispose nr-axSpA patients with normal serum CRP to radiographic progression. We classified the 103 patients with nr-axSpA into groups with either normal or increased CRP levels. Patients with increased serum CRP levels had higher ASDAS-CRP levels (*p* < 0.001) and also higher ASDAS ESR scores (*p* < 0.001). There was a trend but no significant association between serum CRP level and BASDAI scores (*p* = 0.08) (data not shown). We classified the cohort into HLA-B27-positive and -negative groups and demonstrated the same correlation in each group (data not shown). In addition, serum CRP or ESR level was not associated with the presence of BME, SPARCC scores, or structural damage, including sclerosis, erosions, and fJSW (data not shown).

### Difference in the severity of inflammation and structural damage between HLA-B27 positive and negative nr-axSpA

We classified the 103 nr-axSpA patients into two groups: HLA-B27-positive (n = 42) and -negative (n = 61). A higher prevalence and greater severity of BME were noted in HLA-B27-positive patients than in HLA-B27-negative patients (*p* = 0.049 and 0.006, respectively). We analyzed whether inflammation was correlated with structural damage in each group. Among HLA-B27-positive patients, more severe BME was identified in patients with sclerosis on SIJ MRI (*p* = 0.001). In both HLA-B27-positive and -negative groups, BME was more common in the presence of fJSW (phi scores of 0.370 and 0.377, *p* = 0.018 and 0.003, respectively). BME was significantly more prevalent in triple-positive patients with sclerosis, erosions, and fJSW (*p* = 0.018 and 0.012, respectively) (Table 5). In the HLA-B27-positive group, higher SPARCC scores were noted in patients with sclerosis (*p* = 0.002). In both HLA-B27-positive and -negative groups, SPARCC scores were higher in patients with fJSW (*p* < 0.001 and *p* = 0.005). SPARCC scores were significantly higher for both HLA-B27-positive and -negative patients who were triple-positive for sclerosis, erosions, and fJSW (*p* = 0.001 and 0.005, respectively) (Table 4).

**Table 5:** Associations and strengths of the presence of BME and structural damage on MRI-SIJs in HLA-B27( +) and HLA-B27(−) nr-axSpA subjects, respectively (Pearson Chi-Square & phi scores)

Structural damage	BME ( +) vs. BME (–)	*p* value	phi scores
HLA-B27( +) n = 42			
Sclerosis ( +)	65.4% vs. 34.6%	0.001**	0.503
Erosion ( +)	100% vs. 0%	NA	NA
fJSW ( +)	75% vs. 25%	0.018*	0.370
DP^a^	57.1% vs. 42.9%	0.318	0.156
TP^b^	75% vs. 25%	0.018*	0.370
Abnormal serum CRP	35.7% vs. 64.3%	0.429	0.100
Abnormal serum ESR	63.6% vs. 36.4%	0.179	0.210
HLA-B27(–) n = 61			
Sclerosis ( +)	32.4% vs. 67.6%	0.337	0.122
Erosion ( +)	27.6% vs. 72.4%	0.911	0.014
fJSW ( +)	63.6% vs. 36.4%	0.003**	0.377
DP^a^	22.7% vs. 77.3%	0.539	− 0.078
TP^b^	60% vs. 40%	0.012*	0.320
Abnormal serum CRP	54.5% vs. 45.5%	0.524	0.100
Abnormal serum ESR	31.3% vs. 68.7%	0.690	0.051

*BME* bone marrow edema, *MRI* magnetic resonance imaging, *SIJ* sacroiliac joint, *nr-axSpA* nonradiographic spondyloarthritis, *fJSW* focal joint space widening, *CRP* C reactive protein, *ESR* erythrocyte sedimentation rate, *NA* not applicable

^a^DP, double positive, dual presence of sclerosis and erosion on MRI-SIJs;

^b^TP, triple positive, simultaneous presence of sclerosis, erosion, and fJSW on SIJ MRI

*p value < 0.05

**p value < 0.01; phi scores were used for association measure between binary variables

In the HLA-B27-positive group, patients with increased serum CRP levels had significantly higher BASDAI scores compared with those with normal serum CRP levels (*p* = 0.016). In both HLA-B27-positive and -negative groups, patients with increased CRP levels had higher ASDAS-CRP scores (both *p* < 0.001, respectively). In the HLA-B27-positive group, patients with fJSW had significantly higher ASDAS-CRP scores (*p* = 0.034). In the HLA-B27-negative group, patients with fJSW had higher BASDAI scores (*p* = 0.028). In the HLA-B27-positive group, patients triple-positive for sclerosis, erosions, and fJSW had significantly higher ASDAS-CRP scores (*p* = 0.034) (Table 4).

## Discussion

Although published data indicate that structural lesions visible on SIJ MRI are relevant to axSpA, it is debated whether structural lesions could be used for the diagnosis of axSpA because BME is not always seen on MRI [10, 11, 44–46]. In addition, the incidence of HLA-B27 is lower in patients of Asian ethnicity, which makes it more difficult to identify nr-axSpA. Thus, it is critical to clarify the definition of a positive MRI and whether we can utilize structural damage detected on SIJ MRI to diagnose axSpA. In the present study, we aimed to determine the best imaging methodology to diagnose nr-axSpA accurately in the presence or absence of BME on SIJ MRI and in the absence of HLA-B27, because both BME and HLA-B27 are less common in Asia than in Western countries. Our data first demonstrated that the presence of severe bone erosions (fJSW) on SIJ MRI is significantly correlated with the presence and the severity of BME in patients with nr-axSpA that meets the ASAS definition. We also demonstrated a moderate positive correlation between fJSW and the presence or the severity of BME in our entire cohort of patients with clinically suspected nr-axSpA. The simultaneous appearance of sclerosis, erosion, and fJSW in patients was positively correlated with BME on SIJ MRI, which supports our hypothesis that structural damage is related to the detection of BME on SIJ MRI. The AUC for fJSW on SIJ MRI had a fair discriminatory performance. Because BME can be found in healthy people, running enthusiasts, and women postpartum, and is not specific to patients with axSpA [20–22], we recommend that fJSW is used as an additional imaging criterion for diagnosing nr-axSpA, especially in Asian people, who are characterized by a lower prevalence of BME.

There are no specific biomarkers for the severity of nr-axSpA, though the serum levels of ESR and CRP are the most commonly used in AS [47, 48]. Indeed, all nr-axSpA patients in our cohort with increased CRP levels had greater disease activity, such as ASDAS-CRP and ASDAS ESR, compared with those with normal CRP. We showed similar positive correlations between abnormal CRP level and disease activity in both HLA-B27-positive and HLA-B27-negative patients. In clinical experience, we have noticed normal serum CRP and ESR levels among patients with radiographic axSpA or nr-axSpA, which challenges the accurate diagnosis of SpA, regardless of the presence or absence of HLA-B27. Our cohort showed that patients with nr-axSpA who have either normal serum CRP or ESR may still have BME or fJSW on SIJ MRI. Among patients with nr-axSpA and normal serum CRP levels, we demonstrated higher SPARCC scores, higher BASDAI scores, and ASDAS-CRP scores in HLA-B27-positive patients with nr-axSpA, than in patients who were HLA-B27 negative. We also identified a significantly positive correlation between fJSW and BME in patients with nr-axSpA and normal CRP levels, which indicates that we can still apply fJSW to the identification of nr-axSpA patients with normal serum CRP levels, and that the presence of severe structural damage contributes to accurate and early diagnosis of nr-axSpA. Our data help identify structural damage in nr-axSpA with normal serum ESR or CRP levels and indicate that combining imaging techniques and traditional serum biomarkers can contribute to demonstrating inflammation and structural damage at the SIJs of patients with nr-axSpA. Future studies might include genomic profiling, investigation of the intestinal microbiome and metabolomics to provide precise biomarkers, because the current markers ESR and CRP seem inadequate for accurate and early diagnosis [47, 48].

Our data demonstrated that HLA-B27-positive patients with nr-axSpA not only had a higher prevalence of BME, but also more severe BME on SIJ MRI than patients who were HLA-B27 negative. Our observation of fJSW in the absence of BME on SIJ MRI was able to confirm nr-axSpA in patients who were HLA-B27 positive. We demonstrated that fJSW contributes to accurate early diagnosis of nr-axSpA in patients without HLA-B27, because there is a moderate correlation between fJSW and BME. For patients with clinically suspected nr-axSpA who do not carry HLA-B27, have radiographic changes on the plain films and detectable BME on SIJ MRI, we can still use fJSW on SIJ MRI to confirm the diagnosis. There were similar positive correlations between fJSW and BME in HLA-B27-positive and -negative groups, which indicates that using fJSW as a diagnostic criterion is not influenced by the presence of absence of HLA-B27 and is a useful additional tool for diagnosis of nr-axSpA. Together, these findings indicate that bone erosion-related fJSW could be an additional imaging criterion to indicate nr-axSpA on MRI, either in the presence or absence of HLA-B27, and may be useful in an area of low prevalence of HLA-B27 [49]. Definition and quantitation of the severity of fJSW at SIJs in patients with nr-axSpA using CT are scarce, but would be desirable because we know that the resolution of MRI is too limited to determine precisely the severity of fJSW.

There are some limitations to our study. First, it is difficult to determine the precise severity of fJSW using MRI. In the future, we will apply a combination of MRI and CT scans of SIJs to determine the extent of fJSW for accurate diagnosis of nr-axSpA. Second, based on published data [20], it is even difficult to identify normal joints in healthy controls, because healthy control individuals may still have BME and structural damage [22]. Rheumatologists still do not fully understand how to determine whether BME and structural changes are associated with SpA in the presence or absence of radiographic features. In our cohort, we excluded people with weight-bearing issues, postpartum, and old age effects, which may have biased our data and also explained why this study did not enroll healthy people as a control group. Most importantly, people without IBP are rarely diagnosed with axSpA and rarely undergo SIJ MRI, which would facilitate accurate diagnosis in our study. In addition, it is still debated whether nonradiographic and radiographic axSpA are two different diseases or whether nonradiographic SpA is just the early manifestation of radiographic SpA [50, 51]. Long-term MRI follow-up of SIJs is warranted, especially to follow up the structural damage; this is ongoing in our study.

In conclusion, we present data indicating that structural damage such as severe bone erosion-related fJSW on MRI is significantly correlated with the development and the severity of inflammation detected on MRI in those who have clinical IBP and nr-axSpA, and can be used as a diagnostic tool for nr-axSpA, especially in HLA-B27-negative patients who have normal serum ESR and CRP levels.

## Data Availability

All the data is underlying the present study from Tri-Service General Hospital, Taipei, Taiwan.

## Abbreviations

BME: Bone marrow edema
HLA-B27: Human leukocyte antigen-B27
fJSW: Focal joint space widening
SpAs: Spondyloarthropathies
axSpA: Axial SpA
IBD: Bowel disease
IBP: Inflammatory back pain
AS: Ankylosing spondylitis
nr-axSpA: Nonradiographic axSpA
SIJs: Sacroiliac joints
ASAS: Assessment of Spondyloarthritis international Society
MRI: Magnetic resonance imaging
BME: Bone marrow edema
CT: Computed tomography
CRP: C-reactive protein
ESR: Erythrocyte sedimentation rate
BASDAI: Ankylosing Spondylitis Disease Activity Index
ASDAS: Ankylosing Spondylitis Disease Activity Scores
